# Synergistic Effects between mTOR Complex 1/2 and Glycolysis Inhibitors in Non-Small-Cell Lung Carcinoma Cells

**DOI:** 10.1371/journal.pone.0132880

**Published:** 2015-07-15

**Authors:** Suhua Jiang, Zhengzhi Zou, Peipei Nie, Ruiling Wen, Yingying Xiao, Jun Tang

**Affiliations:** 1 Department of Oncology, the 2nd Affiliated Hospital of Guangzhou Medical University, Guangzhou, China; 2 KingMed Diagnostics and KingMed School of Laboratory Medicine, Guangzhou Medical University, Guangzhou, China; Central South University, CHINA

## Abstract

Cancer metabolism has greatly interested researchers. Mammalian target of rapamycin (mTOR) is dysregulated in a variety of cancers and considered to be an appealing therapeutic target. It has been proven that growth factor signal, mediated by mTOR complex 1 (mTORC1), drives cancer metabolism by regulating key enzymes in metabolic pathways. However, the role of mTORC2 in cancer metabolism has not been thoroughly investigated. In this study, by employing automated spectrophotometry, we found the level of glucose uptake was decreased in non-small-cell lung carcinoma (NSCLC) A549, PC-9 and SK-MES-1 cells treated with rapamycin or siRNA against Raptor, indicating that the inhibition of mTORC1 attenuated glycolytic metabolism in NSCLC cells. Moreover, the inhibition of AKT reduced glucose uptake in the cells as well, suggesting the involvement of AKT pathway in mTORC1 mediated glycolytic metabolism. Furthermore, our results showed a significant decrease in glucose uptake in rictor down-regulated NSCLC cells, implying a critical role of mTORC2 in NSCLC cell glycolysis. In addition, the experiments for MTT, ATP, and clonogenic assays demonstrated a reduction in cell proliferation, cell viability, and colony forming ability in mTOR inhibiting NSCLC cells. Interestingly, the combined application of mTORC1/2 inhibitors and glycolysis inhibitor not only suppressed the cell proliferation and colony formation, but also induced cell apoptosis, and such an effect of the combined application was stronger than that caused by mTORC1/2 inhibitors alone. In conclusion, this study reports a novel effect of mTORC2 on NSCLC cell metabolism, and reveals the synergistic effects between mTOR complex 1/2 and glycolysis inhibitors, suggesting that the combined application of mTORC1/2 and glycolysis inhibitors may be a new promising approach to treat NSCLC.

## Introduction

Cancer cells depend on metabolic transformation to maintain proliferation. Commonly, two types of metabolism are found in cancer cells, which are glycolysis with generation of lactate and reduced mitochondrial oxidative phosphorylation metabolism[1]. Cancer cells are able to by pass mitochondrial oxidative phosphorylation, and instead utilize glucose for the macromolecule synthesis for daughter cells[2]. They also convert most of pyruvate (a terminal product of glycolysis), which is supposed to entry into mitochondria, and transformed into lactate through largely unknown mechanism[3]. Increase of both glucose uptake and lactate production is an important hallmark of cancer metabolism. This remarkable metabolic reprogramming, known as the Warburg effect, provides cancer cells an advantage to grow even in regions with hypoxia[4]. Thereby, the especial dependence of cancer cells on glycolysis makes them vulnerable to therapeutic intervention with specific glycolysis target inhibitors[5,6,7].

Although the Warburg effect is a well-recognized hallmark of cancer metabolism, its regulatory mechanism is still largely unclear. The mammalian target of rapamycin (mTOR) is a well conserved serine/threonine kinase, which is the catalytic subunit of two molecular complexes of mTOR complex 1 (mTORC1) and mTOR complex 2 (mTORC2)[8]. mTORC1 contains mTOR, PRAS40, mLST8, and Raptor; while the functionally distinct mTORC2 comprises mTOR, mSIN1, PROTOR, mLST8, and the unique regulatory proteins Rictor[9]. mTORC1 regulates transcription and translation in response to nutrient levels, growth factors and cytokines via phosphorylation of the p70 S6 kinase (p70S6K) and the initiation factor 4E-binding protein 1 (4EBP1), playing vital roles in cell growth, autophagy, and metabolism[10,11,12]. Additionally, mTORC1 is strongly sensitive to the inhibition of naturally occurring compound rapamycin[13,14]. However, the function of mTORC2 remains largely uncharacterized. mTORC1 signaling pathway emerges as a key regulator complex of cellular metabolism in various cancers[11]. Recent studies indicate that mTORC1 plays a key role in regulating glucose uptake, glycolysis, and de novo lipid biosynthesis in cancer cells[15,16]. Growth factor signaling regulated by mTORC1 drives metabolism of cancer cells by mediating expression of key enzymes in metabolic pathways[17]. Among numerous mTORC1 effectors, the Myc family and hypoxia-inducible factors (HIFs) are often activated in various cancers and have been considered to confer metabolic advantages to cancer cells by enhancing the Warburg effect through transcriptional activation of glycolytic enzymes[18]. Importantly, several studies have indicated that siRNA against one of mTORC2 components decreases glucose uptake and lipid metabolism in muscle cells and fat cells[19,20]. However, mTORC2 has not been thoroughly investigated in the metabolism of cancer cells.

In the present study, both mTORC1 and mTORC2 were found to be regulators of glycolytic metabolism in non-small-cell lung cancer (NSCLC) cells, and the inhibition of either mTORC1 or mTORC2 decreased the cell glucose uptake. Furthermore, we found that mTORC1 regulated glycolytic metabolism involving AKT signaling pathway, and NSCLC cell death induced by the inhibition of mTORC1 and mTORC2 was significantly enhanced by glycolytic inhibition. Taken together, these accumulating data may lead to the application of a novel NSCLC therapeutics targeting both mTORC1/2 and glycolytic metabolism.

## Results

### Inhibition of mTOR activation decreased cell viability in NSCLC cells

**Fig 1:**
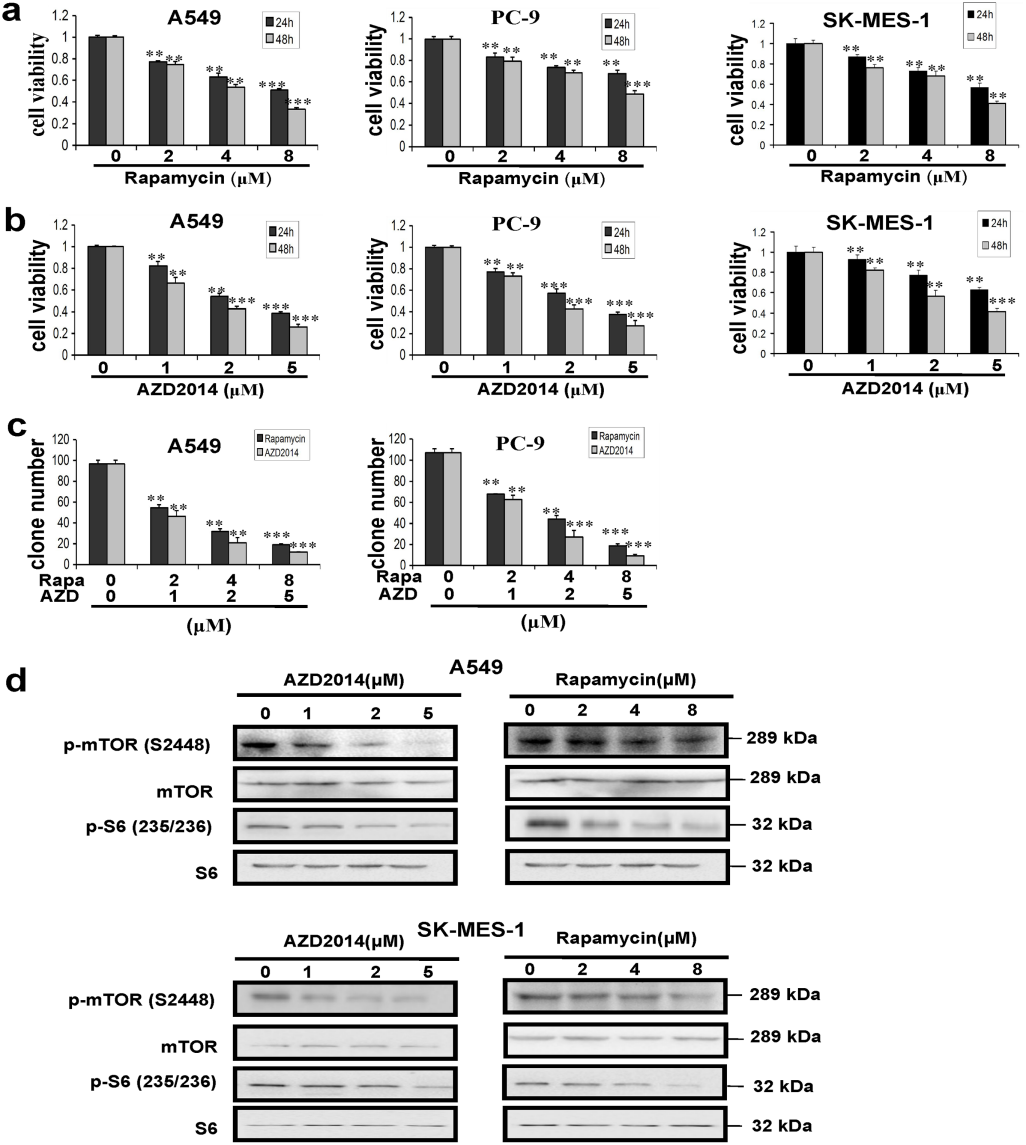
AZD2014 and rapamycin inhibited cell proliferation, colony formation and mTOR signaling in lung cancer cells. Inhibitory effects of AZD2014 and rapamycin on cell proliferation in A549, PC-9 and SK-MES-1 cells (a, b). (c) Influence of AZD2014 and rapamycin on colony-forming ability of A549 and PC-9 cells, evaluated by clonogenic assay. (d) AZD2014 and rapamycin inhibited mTOR signaling in A549 and SK-MES-1 cells as shown by the decreased phosphorylation of mTORand S6 after treatment for 24h. Cell viability was assessed by MTT, and Colony-forming assay was done as described in Materials and Methods. Columns, mean of three determinations; bars, SD. Results shown are representative of three independent experiments. **, *P*< 0.01; ***, *P* < 0.001, control versus AZD2014 or rapamycin-treated cells.

Rapamycin and AZD2014 are inhibitors of mTOR. Rapamycin is the specific inhibitor of mTORC1, while AZD2014 inhibits both mTORC1 and mTORC2. To evaluate the anti-proliferative effects of different doses of mTOR inhibitors, we performed an MTT assay in NSCLC cell lines, A549, PC-9 and SK-MES-1 cells. Treatment with mTORC1 inhibitor rapamycin (ranging from 0, 2, 4 and 8 μM) for 24 h and 48 h resulted in a dose-dependent inhibition of growth with IC_50_ values between 7–7.5 μM and 4–4.5 μM in A549 cells, 15.5–17 μM and 8–8.5 μM in PC-9 cells, as well as 9–9.5 μM and 7–7.5 μM in SK-MES-1 cells at 24 h and 48 h, respectively (Fig 1a). Additionally, A549 cells treated with mTORC1/C2 inhibitor AZD2014 (ranging from 0, 1, 2 and 5 μM) showed IC_50_ values at 1.8–2.4 μM at 24 h and 1.4–1.8 μM at 48 h (Fig 1b, right), PC-9 cells exhibited 2.7–3.2 μM and 1.3–1.7 μM at 24 h and 48 h, respectively (Fig 1b, middle); While SK-MES-1 cells exhibited 5.2–5.7 μM and 2.3–2.8 μM at 24 h and 48 h, respectively(Fig 1b, right). Furthermore, A549 and PC-9 cells exposed to rapamycin or AZD2014 were evaluated for their clonogenic potential. As shown in Fig 1c, both rapamycin and AZD2014 reduced the clonogenic survival of A549 and PC-9 cells in a dose-dependent manner. For example, clone numbers of A549 cells treated with 8 μM rapamycin or 5 μM AZD2014 were reduced to 18.67%or 11.67% as compared with untreated cells (Fig 1c, left).

To confirm the activity of mTOR inhibitors, A549 and SK-MES-1 cells were treated with different concentrations of AZD2014 or rapamycin for 24 h, and then detected for the phosphorylation of mTOR (p-mTOR) and S6 (p-S6) by Western blotting. Both AZD2014 and rapamycin decreased the level of p-mTOR in the cells in a dose-dependent manner (Fig 1d), the level of phosphorylated S6 was obviously reduced in A549 and SK-MES-1 cells treated with either rapamycin or AZD2014 for 24 h.

### Decrease in the level of glycolytic metabolism by inhibition of mTOR in NSCLC cells

**Fig 2:**
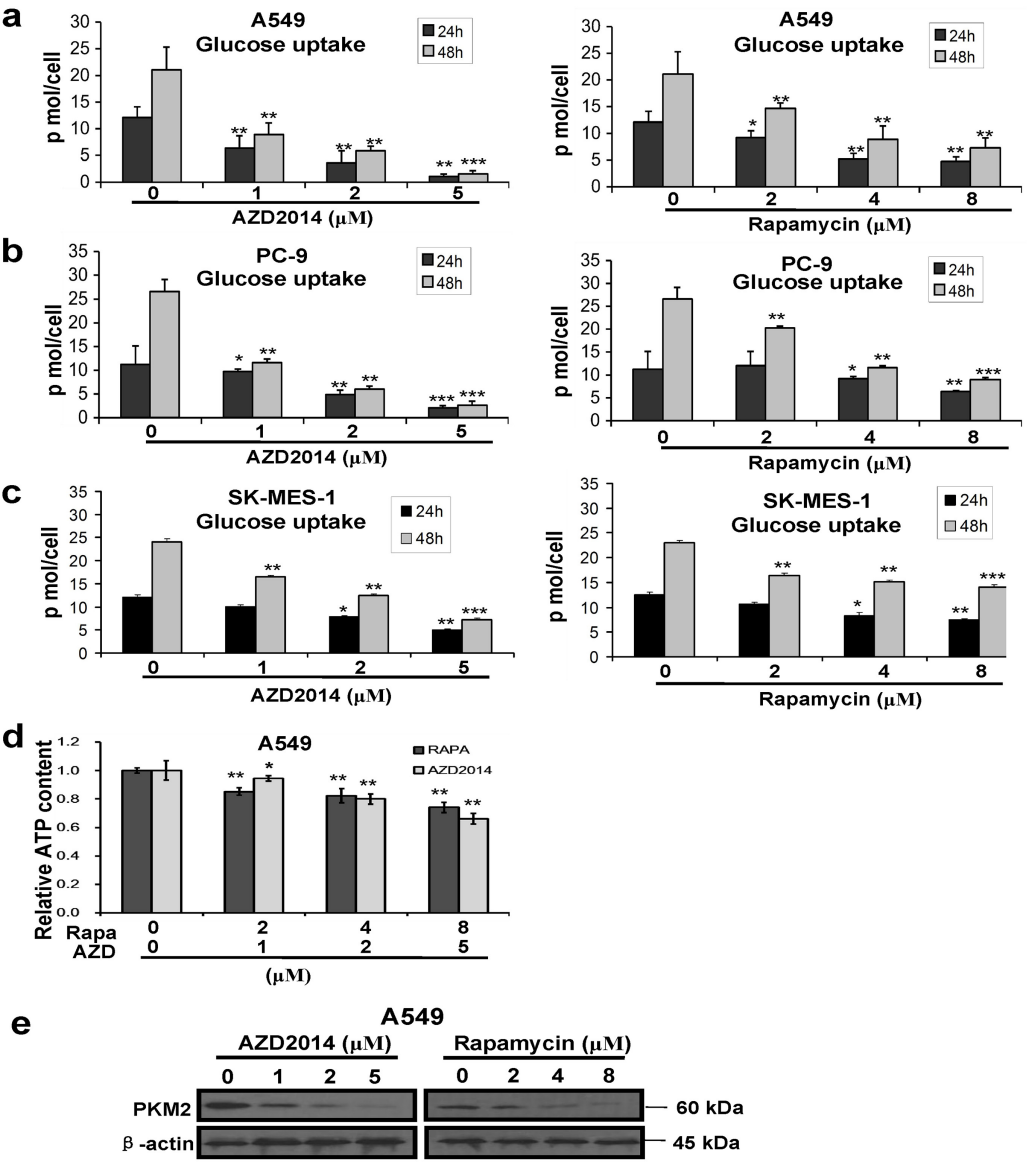
Inhibition of mTOR pathway decreased the level of glycolytic metabolism in lung cancer cells. (a) A549 cells were treated with AZD2014 and rapamycin. (b) PC-9 cells were treated with AZD2014 and rapamycin. (c) SK-MES-1 cells were treated with AZD2014 and rapamycin. After 24 h, the cells were counted and the glucose in the culture media was immediately tested. Then the results were normalized to the number of cells, and performed as pmol/cell. Data represent mean ± SD (n = 3). *P<0.05; **P<0.01; ***P<0.001; control versus AZD2014- or rapamycin-treated cells. (d) and (e) A549 cells were treated with AZD2014 and rapamycin. After 24 h, relative ATP content was determined by using a bioluminescence assay. Then the results were normalized to the number of cells. Data represent mean ± SD (n = 3). *P<0.05; **P<0.01; control versus AZD2014- or rapamycin-treated cells (see Fig 2d). Western blot analysis showed that AZD2014 and rapamycin decreased the level of PKM2 protein in a dose dependent manner. Three independent experiments were performed with similar results, and representative data are shown (see Fig 2e).

In addition to promoting cell growth, mTOR activation could drive specific metabolic processes. A549, PC-9 and SK-MES-1 cells were treated with different concentrations of rapamycin at 0, 2, 4, and 8 μM or AZD2014 at 0, 1, 2, and 5 μM, respectively, and measured for glucose uptake at 24 h and 48 h. As shown in Fig 2a, 2b and 2c, both rapamycin and AZD2014 inhibited the glucose uptake significantly in A549, PC-9, and SK-MES-1 cells in a dose-dependent manner. In addition, glucose uptake activity was measured using a fluorescent D-glucoseanalogue2-[N (7-nitrobenz-2-oxa-1,3-diazol-4-yl)amino]-2-deoxyglucose(2-NBDG) in A549 cells. As shown in S2 Fig, Glucose uptake was decreased significantly in A549 cells treated with different concentrations of AZD2014. In order to investigate the mechanism of glucose metabolism affected by AZD2014, we next detected glucose transporter Glut-1 by immunofluorescence assay. As shown in S3 Fig, the expression of Glut 1 was decreased on the membrane of A549 and PC-9 cells treated with AZD2014 for 48 h, suggesting that the glycolysis metabolism reduction in NSCLC caused by mTORC inhibitor maybe through downregulating glucose transporter Glut1 expression on the cell membrane. ATP assay was performed as well after A549 cells were treated with different concentrations of rapamycin or AZD2014 for 24 h. As shown in Fig 2d, both rapamycin and AZD2014 decreased the ATP content significantly in a dose-dependent manner, with 74.11% and 66.10% of relative ATP contents in the cells treated by the highest concentration of rapamycin and AZD2014, respectively, as compared with controls. In order to further study the mechanism of glycolysis in NSCLC regulated by mTOR, we examined pyruvate kinase isoenzyme type M2 (PKM2), a key player in promoting cancer metabolism by using Western blotting. As shown in Fig 2e, the inhibition of mTOR by rapamycin or AZD2014 reduced PKM2 protein level in A549 cells, and 5 μM AZD2014 or 8 μM rapamycin resulted in very faint bands of PKM2.

**Fig 3:**
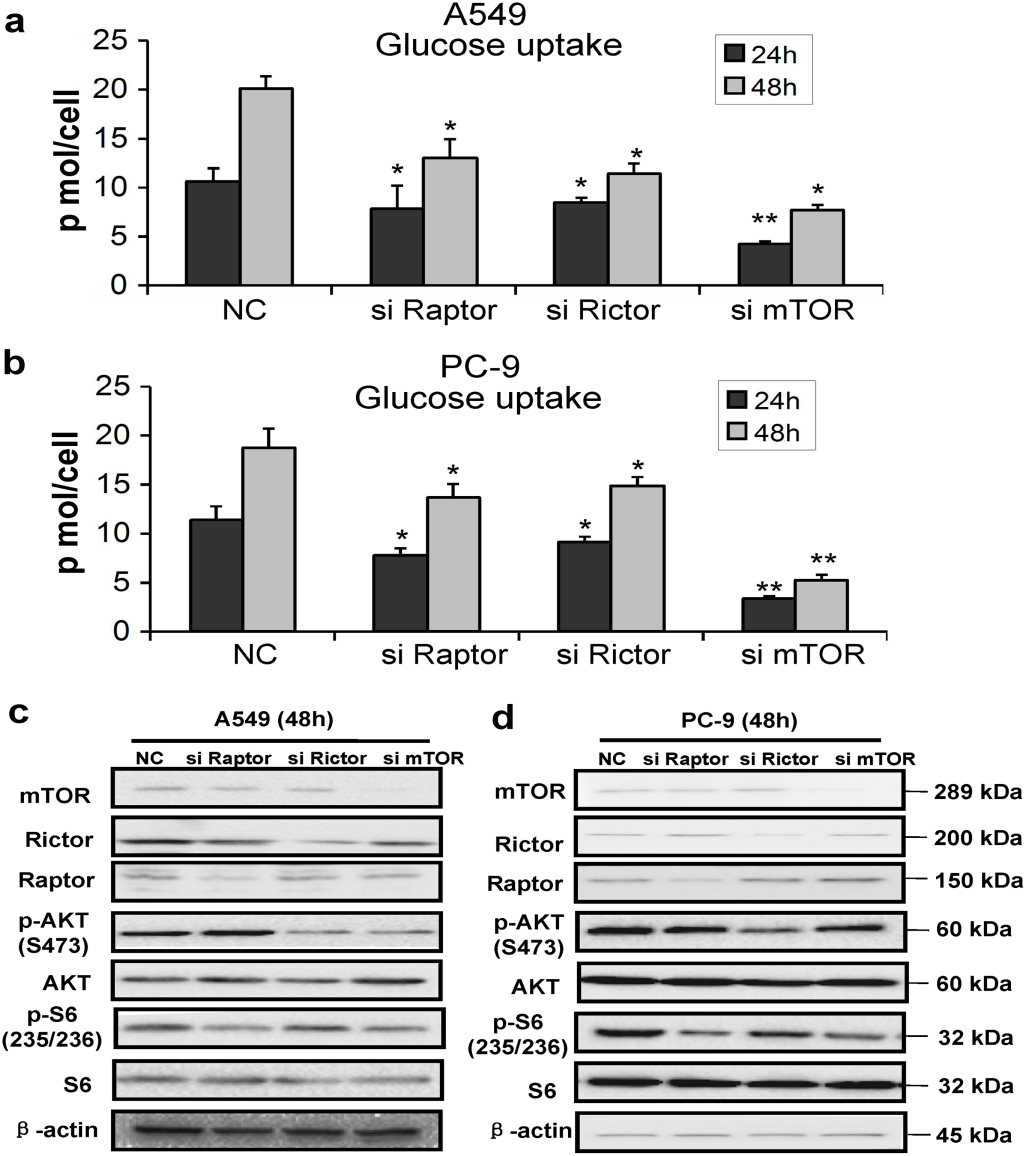
Knockdown of mTOR, Raptor and Rictor decreased the level of glycolytic metabolism in lung cancer cells. (a) and (b) A549 and PC-9 cells were treated with negative control (NC) siRNA or one of those siRNAs targeting Raptor, Rictor, and mTOR. After 24 h and 48 h transfection, the cells were counted and the glucose in the culture media was immediately tested. The results were then normalized to the cells number, and performed as pmol/cell. Data represent mean ± SD (n = 3). *P<0.05, **P<0.01. (c) and (d) Western blot analysis showed that siRNAs against Raptor, Rictor, and mTOR decreased the protein expression of Raptor, Rictor, mTOR, AKT, p-AKT, S6, and p-S6, respectively. Three independent experiments were performed with similar results, and representative data are shown.

At the same time, A549 and PC-9 cells were transfected with siRNAs against Raptor, Rictor and mTOR, and negative control (NC) siRNA. The cells were counted and the level of glucose in the culture media was tested at 24 h and 48 h post-transfection with siRNAs. As shown in Fig 3a and 3b, the amount of glucose uptake decreased remarkably in both A549 and PC-9 cells transfected with siRNAs of Raptor, Rictor or mTOR as compared with that of NC-control cells. In addition, the knockdown of target genes by specific siRNAs was confirmed by Western blotting at 48 h after transfection, and the results revealed that at least 50% of the proteins were specifically down-regulated by corresponding siRNAs (Fig 3c and 3d). As shown in Fig 3c and 3d, the silencing of rictor and mTOR decreased basal levels of p-AKT in A549 and PC-9 cells, and the knockdown of raptor and mTOR inhibited phosphorylation of S6 in both A549 and PC-9 cells. The results revealed that the knockdown of raptor, rictor or mTOR down-regulated their downstream proteins, respectively.

### Effects of mTOR inhibitors alone and in combination with 2-DG on NSCLC proliferation and colony forming ability

**Fig 4:**
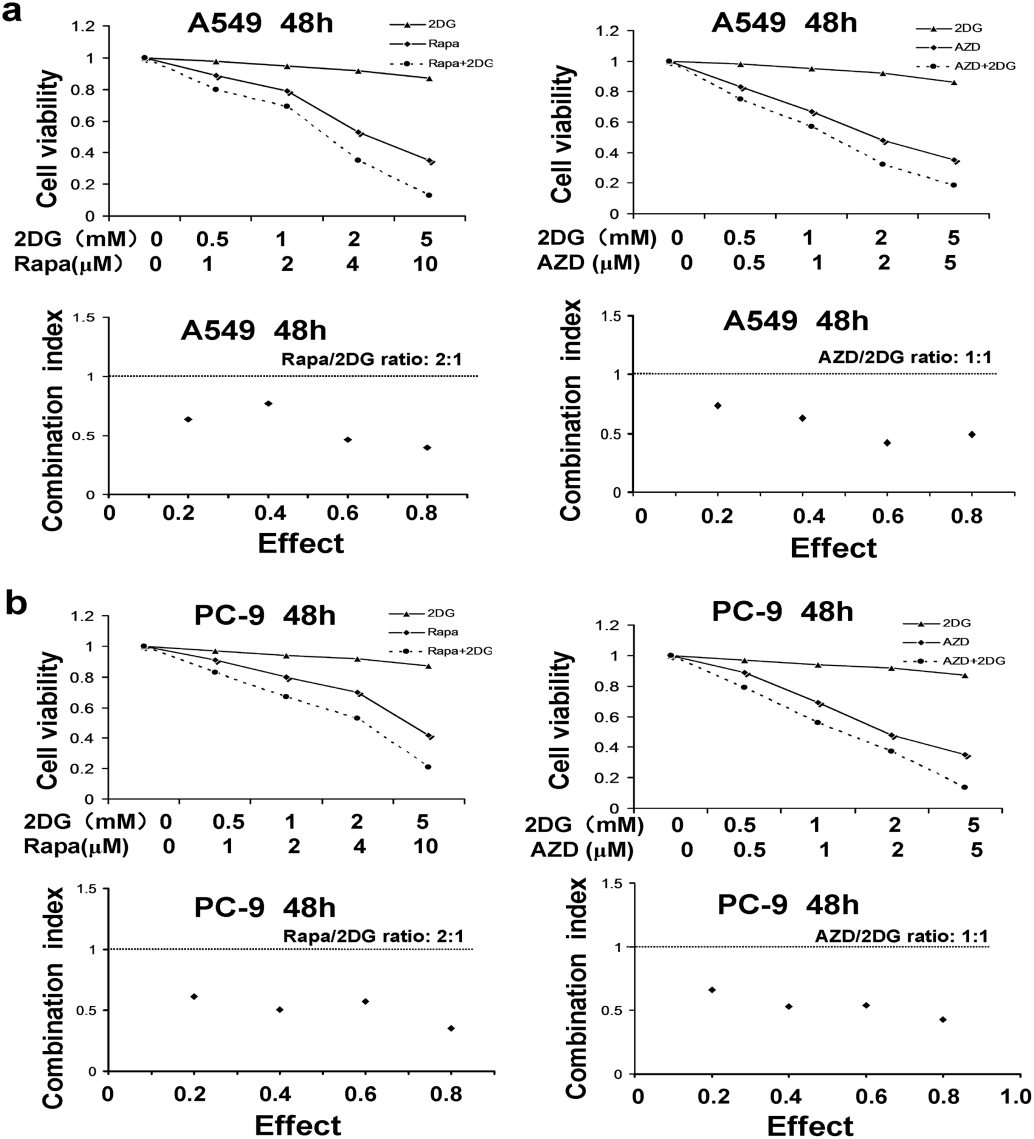
The combination index of mTOR inhibitors and glycolysis inhibitor on cell viability reduction in NSCLC cells. (a) and (b) The dose–response curve of each drug was determined and the combination index (CI) values for rapamycin/2-DG concentration ratios (2:1) and AZD2014/2-DG concentration ratios(1:1) were calculated according to Chou–Talalay’s method at 48 h time point. CI < 1, CI = 1, and CI > 1 indicate synergistic, additive and antagonistic effects, respectively. The effect ranges from 0 (no inhibition) to 1 (complete inhibition). The data are representative of three independent experiments.

**Fig 5:**
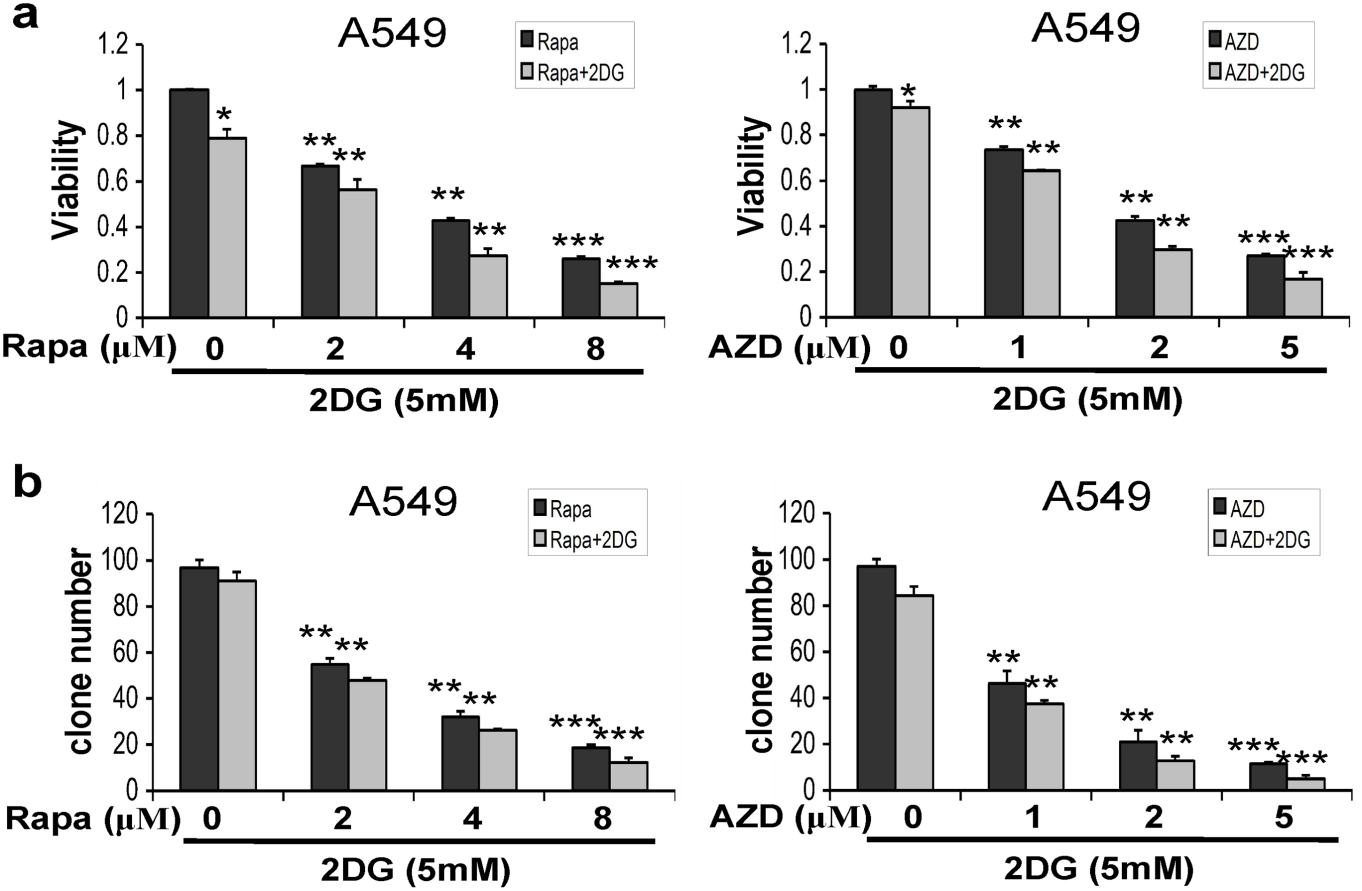
mTOR inhibitors combined with glycolysis inhibitor decreased the colony formation and cell viability in lung cancer cells. (a) Inhibitory effects of rapamycinorAZD2014 combined with 2-DGon cell proliferation in A549 cells. (b) Impact of rapamycinor AZD2014combined with 2-DG on colony forming ability of A549 cells, as evaluated by clonogenic assay. Data represent mean ± SD (n = 3). **, P< 0.01; ***, P < 0.001; control versus AZD2014- or rapamycin-treated cells.

In this study, we found the inhibition of mTOR decreased cell proliferation and anchorage-independent colony forming ability in NSCLC cells. A549 cells were treated with mTOR inhibitor (rapamycin and AZD2014) at different concentrations of 0, 2, 4, 8 μM and 0, 1, 2, 5 μM respectively, combining with 2-DG (5 mM), a glucose analog with the potential for transiently inhibition of glycolysis. Rapamycin, AZD2014, and 2-DG alone reduced cell viability to very limited extents (Fig 4a and 4b), where as a significant loss in cell viability was found in cells treated with either rapamycin or AZD2014 in combination with 5 mM 2-DG compared to that of cells stimulated by rapamycin or AZD2014 alone (Fig 5a). For colony forming ability assay, the inhibition of glycolysis by use of 2-DG (5 mM) resulted in further inhibition of clonogenic survival caused by serial concentrations of mTOR inhibitors, rapamycin or AZD2014 (Fig 5b). These data suggest that rapamycin and 2-DG or AZD2014 and 2-DG may synergize to reduce cell viability in NSCLC cells. To confirm this synergistic possibility, A549 and PC-9 cells were treated with the combination of rapamycin plus 2-DG or AZD2014 plus 2-DG, then the combination index (CI) was calculated using Calcusyn software following Chou-Talalay’s method as previously described[21]. As shown in Fig 4a and 4b, and S4b Fig, the data showed significant synergistic effects between rapamycin and 2-DG or between AZD2014 and 2-DG (CI < 1) in the three NSCLC cells used, but weak synergistic action in normal lung epithelial cells BEAS-2B (S4a Fig). The results suggest that the combined application of mTORC1/2 and glycolysis inhibitors may be a new promising approach to treat NSCLC.

Taken together, these results indicated that the inhibition of glycolysis enhanced cytotoxicity in response to mTOR inhibitors in NSCLC cells.

### Apoptosis induced by mTOR inhibitors combined with glycolysis inhibitor

**Fig 6:**
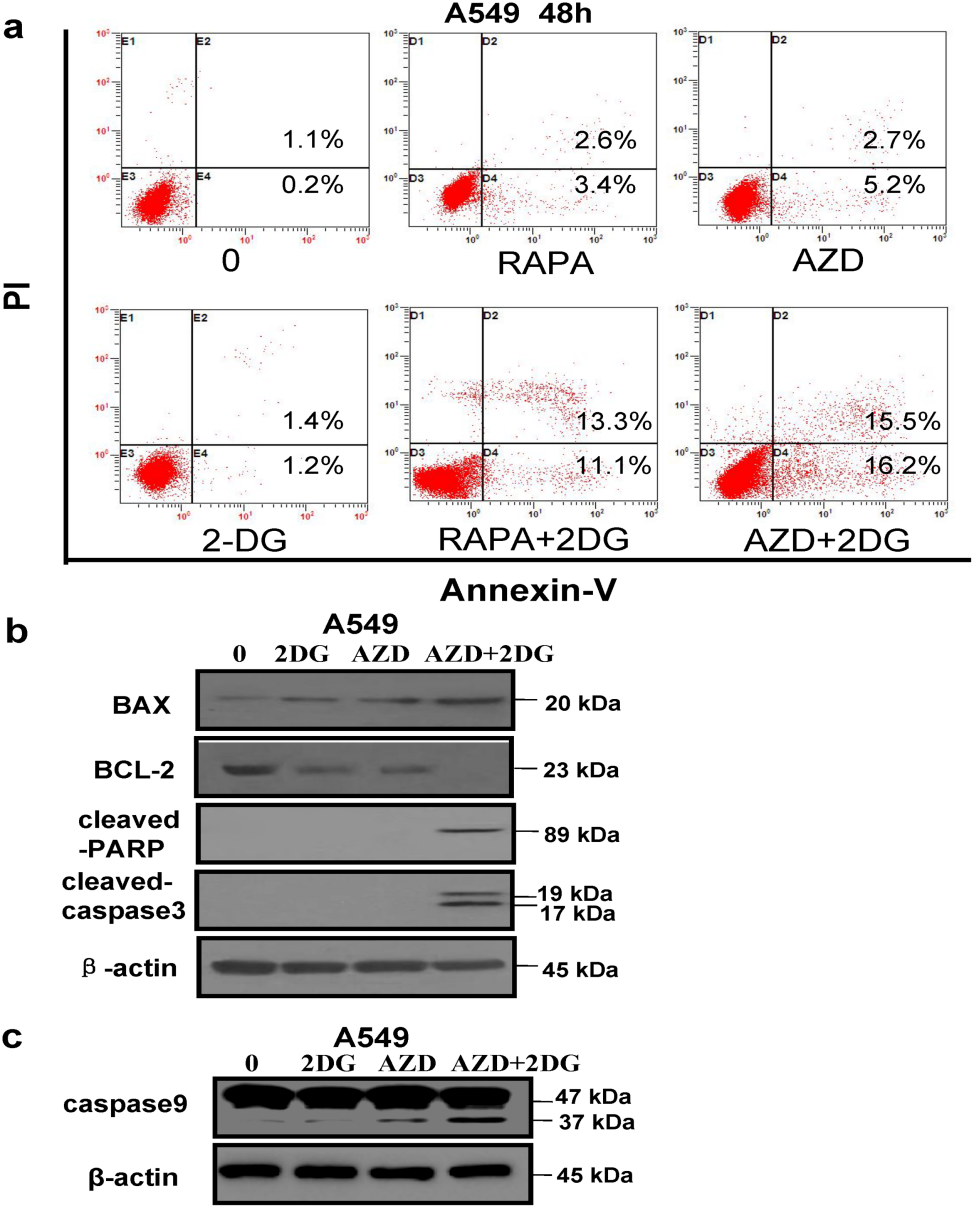
mTOR inhibitors combined with glycolysis inhibitor induced apoptosis in lung cancer cells. (a) A549 cells were pretreated with 5 mM 2DG for 1 h, and then cells were treated with 8 μM rapamycin or 5 μM AZD2014 for additional 48 h respectively, cell apoptosis was then examined by using an Annexin V-FITC/PI staining assay kit. (b) A549 cells were pretreated with 5 mM 2DG for 1 h, and then treated with 5 μM AZD2014 for additional 48 h, as well as the cells were untreated and treated with 5 mM 2-DG or 5 μM AZD2014 alone for 48 h before harvesting cellular proteins. (c) A549 cells were pretreated with 5 mM 2DG for 1 h, and then treated with 5 μM AZD2014 for additional 48 h. Meanwhile, the cells were untreated and treated with 5 mM 2DG or 5 μM AZD2014 alone for 48 h before harvesting cellular proteins. All cell lysates were probed for the apoptosis–associated proteins such as BAX, BCL-2, cleaved PARP, cleaved caspase-3 and caspase 9 by Western blotting. The similar results were obtained from other two independent experiments, and data shown are the representatives of the three experiments.

The data shown above indicated that the inhibition of glycolysis could increase the cytotoxicity of mTOR inhibitors in A549 cells. We then asked if the inhibition of glycolysis could enhance apoptosis induced by mTOR inhibitors in lung cancer cells. We inhibited cell glycolysis by using 2-DG, and then analyzed cell apoptosis induced by rapamycin and AZD2014 using Annexin V-FITC/PI staining in A549 cells. As shown in Fig 6a, the treatment with rapamycin, AZD2014, or 2-DG alone had weak effects on cell apoptosis, and the percentages of apoptotic cells were 2.6%, 2.7%, and 1.4%, respectively. However, there were significantly increased apoptosis rates (13.3% and 15.5% respectively) in the cells treated by the combination of either rapamycin or AZD2014 with 2-DG (Fig 6a). To examine the apoptosis related molecules, such as pro-apoptotic factor BAX, anti-apoptotic protein BCL-2, cleaved caspase 3, and cleaved PARP, we next treated A549 cells with AZD2014 or 2-DG alone, or in the combination and then detected the apoptosis associated proteins by Western blotting. As shown in Fig 6b, 2-DG increased the expression of BAX induced by AZD2014. Conversely, decrease of BCL-2 expression by AZD2014 was further decreased by 2-DG (Fig 6b). In addition, bands for both of cleaved caspase 3 and cleaved PARP were observed in the cells stimulated with combination of AZD2014 and 2-DG, while no band was detected in cells stimulated with AZD2014 or 2-DG alone (Fig 6b). As shown in Fig 6c, caspase-9 was activated in A549 cells after treatment with AZD2014 or AZD2014 combined with 2DG, suggesting the involvement of mitochondrial apoptotic pathway in the apoptosis induced by mTOR inhibitor alone and combined with glycolysis inhibitor in lung cancer cells.

### Downregulation of glycolysis metabolism by mTORC1 inhibitor was enhanced by AKT inhibition in lung cancer cells

**Fig 7:**
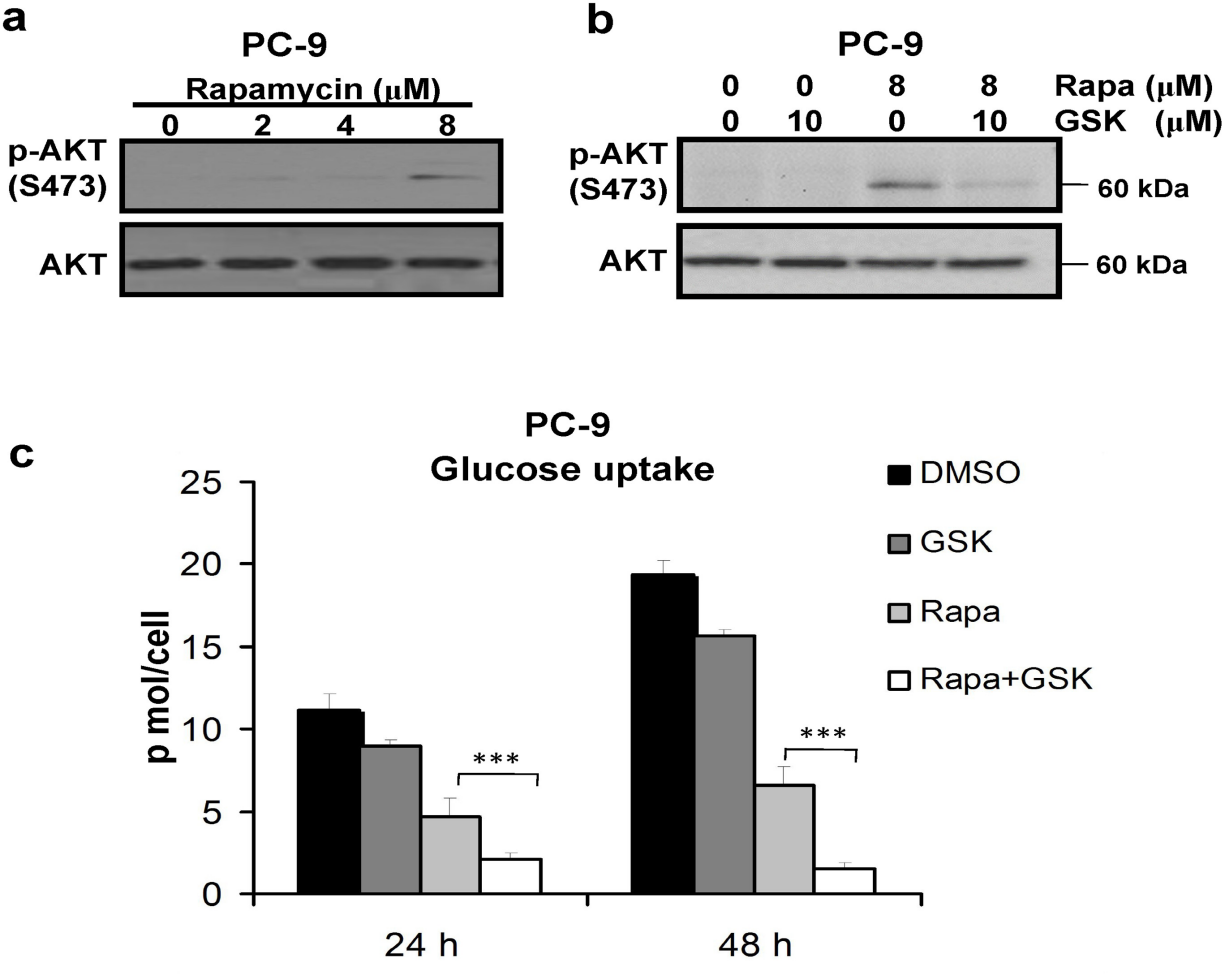
Inhibition of glycolysis metabolism by rapamycin was enhanced by AKT inhibition in PC-9 cells. (a) PC-9 cells were treated with rapamycin for 24 h, and then cell lysates were assayed for p-AKT by Western blotting. (b) PC-9 cells were treated with rapamycin for 24 h and GSK690693 for 1 h respectively, or pre-treated with 10 μM GSK690693 for 1 h and then with rapamycin for 24 h. The cells were probed for p-AKT. (c) Glucose uptake was assayed in PC-9 cells treated with GSK690693, rapamycin, or in the combination. The experiments were repeated for at least three times. Data represent mean ± SD, ***P < 0.001.

It has been suggested that AKT phosphorylation could be enhanced following mTORC1 inhibition. Our findings in Fig 2b showed a significant inhibition of glucose uptake by mTORC1 inhibitor rapamycin, thus we thought AKT may be involved in the regulation of glycolysis metabolism. To further investigate this question, we extended analysis on PC-9 cells using rapamycin and tested AKT activation. As shown in Fig 7a, we found that rapamycin induced AKT phosphorylation in a dose dependent manner in the cells. Given that AKT phosphorylation has been reported to stimulate cell glucose uptake, we next tested if AKT inhibition can further enhance the inhibition of glycolysis metabolism caused by rapamycin in lung cancer cells. PC-9 cells were treated with rapamycin or in combination with GSK90936, a specific AKT inhibitor. As shown in Fig 7b, the inhibition effect of GSK90936 was confirmed by Western blotting. In Fig 7c, PC-9 cells were treated with 8 μM rapamycin alone or in combination with 10 μM GSK90936. The cells treated in the combination with rapamycin and GSK90936 showed glucose uptake inhibition by 57.9 ± 1.1% at 24 h and 76.1 ± 0.9% at 48 h, respectively, compared with the cells treated with rapamycin alone, which indicates the inhibition of cellular glucose uptake mediated by rapamycin was further enhanced by AKT inhibitor GSK90936.

## Discussion

Lung cancer is the firstcause of malignancy related mortality worldwide, with more than one million cases diagnosed yearly[22]. NSCLC, accounting for 80% of all lung cancers, includes squamous carcinoma, adenocarcinoma and large cell carcinoma. Conventional regimen of NSCLC has clearly reached a plateau of effectiveness in increasing the survival rates in patients. Thereby, novel cancer therapeutic methods are pressing. As an important regulator of cell growth and proliferation, the mTOR has been the subject of intense investigation for its role in tumor development and progression[23,24]. Compounds targeting the mTOR pathway have the potential of application in cancer treatment[25]. Such compounds include rapamycin and its derivatives (CCI-779, RAD001, and AP23573) as well as AZD8055 and its derivatives like AZD2014[26,27].

In addition, aerobic glycolysis is considered as an indispensable change acquired by cancer cells to thrive. Therefore, targeting metabolic enzymes is believed to be one of promising anticancer strategies. Drugs that inhibit glycolysis of cancer cells by targeting a variety of molecules are under clinical trials[28]. Moreover, mTORC1 has emerged as a key regulator of cellular metabolism, besides its better-known functions in promoting protein synthesis and cell growth[8]. Recent studies suggest that mTORC1 activation is sufficient to stimulate glucose uptake, glycolysis, and *de novo* lipid biosynthesis, which are considered metabolic hallmarks of cancers[13]. Carolyn et al found that the inhibition of mTORC1 by rapamycin prevented glucose uptake and decreased the expression of GLUT1 in Rat LEF cells[28,29]. Sun et al have shown that hyperactive mTOR promotes aerobic glycolysis by upregulating the expression of PKM2 in MEF cells[30]. Moreover, Arvind et al found that mTOR inhibition can yield tricarboxylic acid (TCA) cycle intermediates to inhibit aerobic glycolysis, and decreased uncoupled mitochondrial respiration[31].

In this study, we found the inhibition of mTORC1 or mTORC1/2 with rapamycin or AZD2014 respectively decreased the levels of glucose uptake and ATP production in NSCLC cells, which indicates that the inhibition of mTORC1 or mTORC1/2 decreased aerobic glycolysis in NSCLC cells.

Notably, mTORC1 has been found to link oncogenic signaling to tumor cell metabolism[16]. mTORC2 has been shown to function as a key regulator of the cytoskeleton through its stimulation of F-actin stress fibers, paxillin, RhoA, Rac1, Cdc42, and protein kinase Cα[32]. In addition, mTORC2 promotes glycolytic metabolism through AKT-independent pathway in glioblastoma and AKT-dependent pathway in mice hepatic cells[33,34]. mTORC2 also plays key role during osteoblast differentiation by AKT pathway[35]. However, the roles of mTORC2 in regulating NSCLC cell metabolism have not been thoroughly identified. In this study, we found that the inhibition of mTORC1 using rapamycin decreased glucose uptake (Fig 2a and 2b). As compared with mTORC1 inhibitor rapamycin, the dual mTORC1/2 inhibitor AZD2014 was able to reduce cellular metabolic levels in A549 and PC-9 cells (Fig 2a). In addition, the decrease in glucose uptake was found in NSCLC cells treated with siRNA against Rictor, an upstream molecule of mTORC2 (Fig 3a and 3b). These studies indicate that mTORC2 regulates glucose metabolism in NSCLC cells. Furthermore, several studies reported that rapamycin could activate AKT signaling pathway through a negative feedback loop while inhibiting mTORC1 signaling[36]. Interestingly, Wang et al reported that the low concentration of rapamycin activates AKT while the high concentration suppresses the phosphorylation of AKT in cancer cells[37]. Additionally, they showed that rapamycin increased AKT phosphorylation through a mechanism independent of mTOR/Rictor[37,38]. Reversely, Madlaina et al found that increased AKT Ser473 phosphorylation induced by RAD001 (everolimus), an mTORC1 (mTOR/Raptor) inhibitor, was Rictor dependent[38]. It has been reported that mTORC2 activates AGC kinase family members, including AKT, protein kinase Cα and SGK1 (serum- and glucocorticoid-induced protein kinase 1)[39]. It is reported that the PI3K/AKT pathway promotes glucose metabolism in many cancers[40]. AKT exerts a direct influence on glycolysis in cancer cells by regulating the localization of GLUT1 to the plasma membrane and regulating hexokinase expression, activity, and mitochondrial interaction[41]. In addition, AKT may indirectly activate the glycolysis rate-controlling enzyme phosphofructokinase-1 (PFK1) by directly phosphorylating phosphofructokinase-2 (PFK2), which produces the product, fructose-2.6-bisphosphate (Fru-1, 6-P2)[42].

In this study, we found that mTORC1 inhibitor rapamycin induced AKT activation (Fig 7a) and rapamycin-mediated cellular glucose uptake inhibition was further decreased by AKT inhibitor GSK90936 (Fig 7c). The metabolic properties of cancer cells are different from those of normal cells, and cancer cells are more dependent on aerobic glycolysis. This distinction suggests that targeting metabolic dependence could be a promising anticancer approach to treat cancer patients. Recently, several reports have shown that metabolic inhibitors induced cell death in cancer cells[5]. Moreover, targeting of metabolic enzymes, such as glucose transporters, hexokinase, PKM2, lactate dehydrogenase A, pyruvate dehydrogenase kinase, fatty acid synthase and glutaminase can enhance the efficacy of common therapeutic agents or overcome resistance to chemotherapy or radiotherapy for cancers[43,44]. In this study, we found that NSCLC cells were significantly more sensitive to mTORC1/2 inhibitor than mTORC1 inhibitor. Moreover, treatment using AZD2014 combined with the cytostatic glycolysis inhibitor 2-DG synergistically and simultaneously induced the inhibition of cell proliferation and apoptosisin NSCLC cells. These results further support the growing body of evidence that the combination application of AZD2014 and 2DG may be a better therapeutic approach for NSCLC than mTORC1/2 inhibitor alone. Moreover, the enhanced caspase-3 activity and Bax expression induced by the combination of AZD2014 with glycolysis inhibitor were found in NSCLC cells. These indicated that mitochondrial apoptotic pathway was involved in cells apoptosis induced by the combination treatments of both mTOR and glycolysis inhibitors.

In conclusion, here we demonstrated that the regulation of glycolysis by mTORC1 inhibitor was involved not only in mTORC1, but also in feedback activation of AKT pathway in NSCLC cells, and that there were the synergistic effects between mTOR complex 1/2 and glycolysis inhibitors, suggesting the application of a glycolysis inhibitor may further enhance the anticancer effect of mTORC1/2 inhibitors in the treatment of NSCLC.

## Materials and Methods

### Cell culture, reagents and drug treatment

A549, PC-9 and SK-MES-1 cell lines were obtained from Cell Bank at the China Academy of Science (Shanghai, China). Normal lung epithelial cell BEAS-2B was obtained from the Infection and Immunity Research Center, Guangzhou Institutes of Biomedicine and Health, Chinese Academy of Sciences. A549, PC-9 and BEAS-2B cells were grown in RPMI 1640 medium (Life Technologies, Carlsbad, CA, USA) supplemented with 10% (v/v) fetal bovine serum (FBS) (Life Technologies, Carlsbad, CA) at 37°C in 5% CO_2_ incubator, and SK-MES-1 cells grown in MEM medium (Corning,USA) supplemented with 10% (v/v) fetal bovine serum (FBS) at 37°C in 5% CO_2_ incubator. Cells were routinely passaged every 2 or 3 days. AZD2014 and GSK690693 were purchased from Selleck Chemicals LLC (Houston, TX, USA). Rapamycin, 2-Deoxyglucose(2-DG),3-(4,5-Dimethylthiazol-2-yl)-2,5-diphenyltetrazolium bromide (MTT), and dimethyl sulfoxide (DMSO) were all purchased from Sigma (St. Louis, MO, USA). For drug reconstitution, AZD2014, rapamycin and GSK690693 were dissolved in DMSO, while 2-Deoxyglucose was dissolved in sterilized distilled water, and they were stored in aliquots at -80°C. Stock solutions were diluted to the desired final concentrations with growth medium priortouse. Cells were seeded in triplicate at 1×10^6^ cells / well into six-well plates and cultured with fresh growth media for at least 12 h, then incubated with fresh media containing different doses of drugs for additional 24 h or 48 h.

### Cell viability, clonogenic cell survival and apoptosis assays

Cell viability was analyzedby a standard MTT assay. The cells were seeded into 96-well plates, starved and stimulated with specific drugs for a specific time. And then MTT (5 mg/ml in phosphate buffered saline) was added to each well and incubated for 4 h prior to discarding the supernatants. Finally, the cells were solubilized in 200 μl of DMSO and the optical density at 490 nm was measured using a multi-well plate reader (Micro-plate Reader; Bio-Rad, Hercules, CA, USA).

For clonogenic cell survival experiments, the attached cells from the same dish were trypsinized with 1 ml trypsin–EDTA (Life Technologies, Carlsbad, CA, USA) and inactivated with media containing 10% FBS. After that, the cells were diluted and counted using a hemocytometer before seeded at 150–200 cells per well into six-well plates. The clones were allowed to grow in a humidified 5% CO_2_, 37°C environment for 15 days in growth medium, and in the presence of specific drugs. The number of cell colonies was counted after fixed by 70% ethanol and stained with crystal violet.

Cell apoptosis was detected by using the Apoptosis Detection Kit (BD Biosciences, San Jose, CA, USA) following the manufacturer’s protocol as described previously[45].

### Western blotting

Cell lysate preparation and Western blotting were performed as previouslydescribed[46]. Specific primary antibodies used for Western blotting included p-mTOR (Ser2448, Cat. No. 5536S), mTOR (Cat. No. 2983S), BAX (Cat. No. 2774S), BCL-2 (Cat. No. 2870S), cleaved caspase 3 (Asp175, Cat. No. 9664S), cleaved PARP (Asp214, Cat. No. 9541S), PKM2 (Cat. No. 3198S), p-AKT (Ser473, Cat. No. 4060S), AKT (Cat. No. 4691S), p-S6 (Cat. No. 4858S), S6 (Ser235/236,Cat. No. 2217S), Rictor (Cat. No. 9476S), Raptor (Cat. No. 2280S), and β-Actin (Cat. No. 4967S) antibodies (purchased from Cell Signaling Technology, Danvers, MA, USA). And Caspase 9 (Cat. No. ab32539) antibody was purchased from Abcam, USA.

### ATP assay

ATP cell content was determined over time using the ATP Bioluminescent Somatic Cell Assay kit (Sigma, St. Louis, MO, USA) according to the manufacturer’s recommendations. Briefly, cells were seeded and treated with specific drugs for 24 h. Afterward, the cells were washed twice with PBS and lysed on ice with Somatic Cell ATP Releasing Reagent. The cell lysis to be assayed and ATP internal standards provided by the kit were then mixed briskly, and transferred to ATP Assay Mix Working Solution containing luciferase and luciferin, followed by immediately measuring the amount of light emitted with a luminometer. The amount of ATP in the cell sample was calculated according to the equation provided by the manufacturer.

### RNA interference

Down-regulation of target gene expression in cells was done by transfection of siRNA oligonucleotides with Lipofectamine 2000 transfection reagent (Life Technologies, Carlsbad, CA, USA) according to the manufacturer’s instructions. A549 or PC-9 cells were plated on a 35-mm culture dish in RPMI-1640 complete growth medium to reach 50% ~ 60% confluence the next day when they were transfected with siRNAs as previously described[45]. The negative control (NC) siRNA and siRNAs against *Raptor*, *Rictor*, and *MTOR* were synthesized by Life Technologies. The sequences of siRNAs for *Raptor*, *Rictor*, and *MTOR* were indicated below: 5'-GGACAACGGCCACAAGUAC-3', 5'-GCGAGCTGATGTAGAATTG-3', and 5'-AACGCGGCACUUCACAAUUGA-3', respectively.

### Measurements of glucose levels in the cell media

The cells were seeded at 3×10^5^ cells per well into a 12-well plate, treated with specific drugs for a specific time, and the number of viable cells was counted after trypan blue staining. For assessment of glucose uptake, the media were collected and the glucose was immediately measured by automated spectrophotometry performed on the Olympus AU5400 system (Olympus Corporation, Tokyo, Japan)[47].

### Immunofluorescence staining of GLUT-1 protein

To detect the expression of GLUT-1 protein in cells, the immunofluorescence technique with specific antibody against GLUT-1 (Cat. No. ab32551, Abcam, USA) was performed. Anti-rabbit Alexa Fluor 488 IgG (Cat. No.ab150077, Invitrogen, USA) was used as the secondary antibody. Photographic images were taken using a fluorescence microscope (Olympus, Tokyo, Japan).

### Measurement of glucose uptake activity using 2-NBDG

Glucose uptake activity was measured using a fluorescent D-glucose analogue 2-[N (7-nitrobenz-2-oxa-1,3-diazol-4-yl)amino]-2-deoxyglucose in A549 cells. Briefly, A549 cells were treated by different concentrations of AZD2014 in a 24-wells plate for 48 h. Then the cells were incubated in glucose-free medium for 30 min, and 60 μM 2-NBDG was then added to the medium for additional 30 min before use. The fluorescence intensity of cellular 2-NBDG was observed under fluorescence microscope. Then the cells were suspended with PBS after trypsinization and transferred to a 96-wells black plate. The cellular fluorescence intensity was measured using Fluorescent microplate reader (Molecular Devices,USA)(excitation wavelength: 485 nm,emission wavelength: 535 nm).

### Combination Index

To confirm synergistic effect of 2-DG combined with rapamycinor AZD2014, CalcuSyn software was used for analyzingthe combination index (CI) in A549 and PC-9 cells. The combination index was calculated using Calcusyn software following Chou-Talalay’smethod[21]. From the analysis, the combination effects of the two agents can be summarized as follows: CI < 1, CI = 1, and CI > 1 indicate synergistic, additive and antagonistic effects, respectively, as described previously [48].

### Statistics

Student’s t tests were conducted usingthe Statistical Package for Social Sciences (SPSS) software (version 16.0) and *P* value < 0.05 considered significant. Every experiment in this study was performed for three times and data were presented as mean ± standard deviation (SD).

## Supporting Information

S1 Fig Effects of rapamycin and AZD2014 on proliferation and glucose uptake in BEAS-2B cells. (a) Inhibitory effects of AZD2014 and rapamycin on BEAS-2B cell proliferation. Cell viability was assessed by MTT. (b) AZD2014 and rapamycin inhibited mTOR signaling in BEAS-2B cells as shown by the decreased phosphorylation of mTOR after treatment for 24h.(c) BEAS-2B cells were treated with AZD2014 and rapamycin. After 24 h, the cells were counted and the glucose in the culture media was immediately tested. Then the results were normalized to the number of cells, and performed as pmol/cell. Data represent mean ± SD (n = 3). *P<0.05; **P<0.01; ***P<0.001; Columns, mean of three determinations; bars, SD. Results shown are representative of three independent experiments. **, P< 0.01; ***, P < 0.001; control versus AZD2014- or rapamycin-treated cells.
(TIF)

S2 Fig Glucose uptake activity was measured using 2-NBDG. (a) The fluorescence intensity of 2-NBDG was observed under fluorescence microscope in A549 cells treated with AZD2014 for 48 h.(b) The cellular fluorescence intensity was measured using fluorescent microplate reader in A549 cells treated with AZD2014 for 48 h.(a) and (b) Cells were incubated with different concentrations of AZD2014 for 48 h. And then, cells were incubated in glucose-free medium for 30 min before 60 μM 2-NBDG was added to the medium for another 30 min as described in Methods. Data represent mean ± SD (n = 3). *P<0.05; **P<0.01; ***P<0.001; Columns, mean of three determinations; bars, SD. Results shown are representative of three independent experiments. **, P< 0.01; ***, P < 0.001; compared to untreated group.
(TIF)

S3 Fig Inhibition of mTOR pathway decreased the level of GLUT1on NSCLC cell membrane. (a) Detection of GLUT1protein in A549 cells treated with 5 μM AZD2014 for 48 h by immunofluorescence assay. (b) Detection of GLUT1protein in PC-9 cells treated with 5μM AZD2014 for 48 h by immunofluorescence assay.
(TIF)

S4 Fig Inhibitory effects of AZD2014 orrapamycin combinedwith 2-DG on cell proliferation in BEAS-2B and SK-MES-1 cells. (a) Inhibitory effects of rapamycin or AZD2014 combined with 2-DG on cell proliferation in BEAS-2B cells. Cells were treated with indicated concentrations of rapamycin, AZD2014 and 2-DG for 48 h. Cell proliferation was assessed by MTT assay. (b) Inhibitory effects of rapamycin or AZD2014 combined with 2-DG on cell proliferation in SK-MES-1 cells. Cells were treated with indicated concentrations of rapamycin, AZD2014 and 2DG for 48 h. Cell proliferation was assessed by MTT assay. (a) and (b) The dose–response curve of each drug was determined and combination index (CI) values for rapamycin/2-DG concentration ratios (2:1) and for AZD2014/2-DG concentration ratios(1:1) were calculated according to the Chou–Talalay’s method at48 h time point. Diamond symbol designates the CI value for each fraction affected (effect). CI < 1, CI = 1, and CI > 1 indicate synergistic, additive and antagonistic effects, respectively. The effect ranges from 0 (no inhibition) to 1 (complete inhibition). The data are representative of three independent experiments.
(TIF)
