# Supplementary figures and images for: Synergistic Effects between mTOR Complex 1/2 and Glycolysis Inhibitors in Non-Small-Cell Lung Carcinoma Cells
Source: PLoS One. 2015 Jul 15;10(7):e0132880. doi: 10.1371/journal.pone.0132880 (PMC4503566; doi:10.1371/journal.pone.0132880)

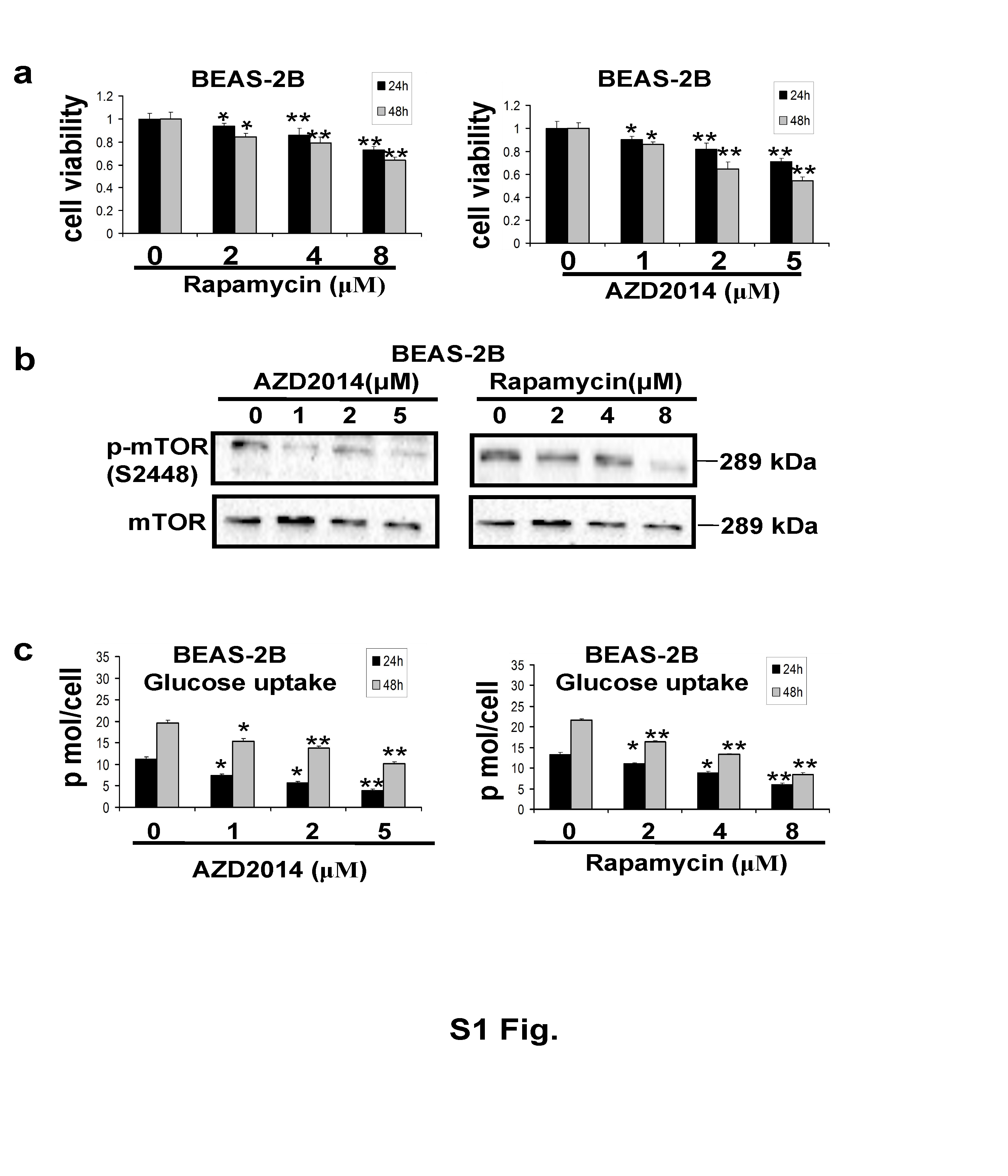

Supplement: S1 Fig — (a) Inhibitory effects of AZD2014 and rapamycin on BEAS-2B cell proliferation. Cell viability was assessed by MTT. (b) AZD2014 and rapamycin inhibited mTOR signaling in BEAS-2B cells as shown by the decreased phosphorylation of mTOR after treatment for 24h.(c) BEAS-2B cells were treated with AZD2014 and rapamycin. After 24 h, the cells were counted and the glucose in the culture media was immediately tested. Then the results were normalized to the number of cells, and performed as pmol/cell. Data represent mean ± SD (n = 3). *P<0.05; **P<0.01; ***P<0.001; Columns, mean of three determinations; bars, SD. Results shown are representative of three independent experiments. **, P< 0.01; ***, P < 0.001; control versus AZD2014- or rapamycin-treated cells. (TIF) [file pone.0132880.s001.tif]

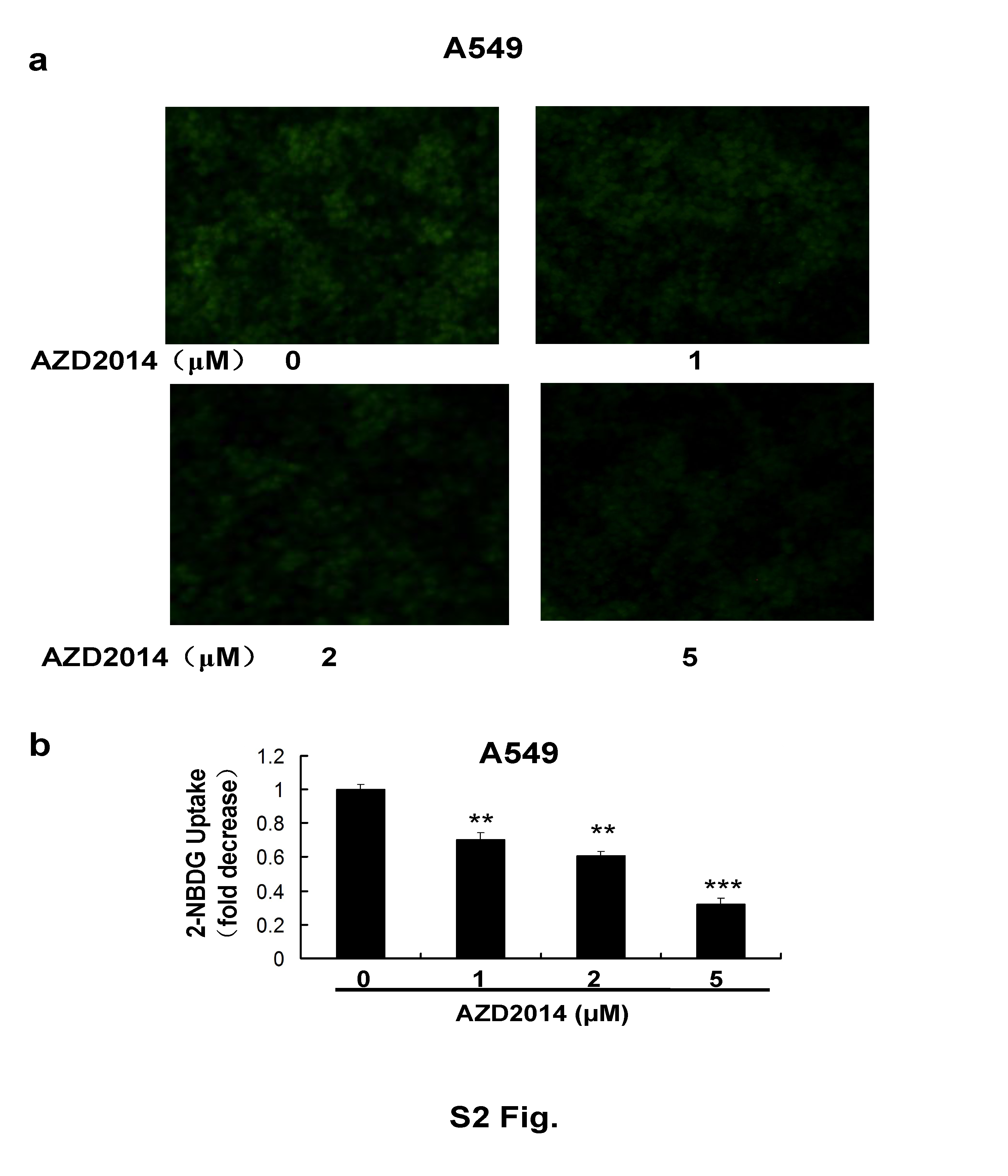

Supplement: S2 Fig — (a) The fluorescence intensity of 2-NBDG was observed under fluorescence microscope in A549 cells treated with AZD2014 for 48 h.(b) The cellular fluorescence intensity was measured using fluorescent microplate reader in A549 cells treated with AZD2014 for 48 h.(a) and (b) Cells were incubated with different concentrations of AZD2014 for 48 h. And then, cells were incubated in glucose-free medium for 30 min before 60 μM 2-NBDG was added to the medium for another 30 min as described in Methods. Data represent mean ± SD (n = 3). *P<0.05; **P<0.01; ***P<0.001; Columns, mean of three determinations; bars, SD. Results shown are representative of three independent experiments. **, P< 0.01; ***, P < 0.001; compared to untreated group. (TIF) [file pone.0132880.s002.tif]

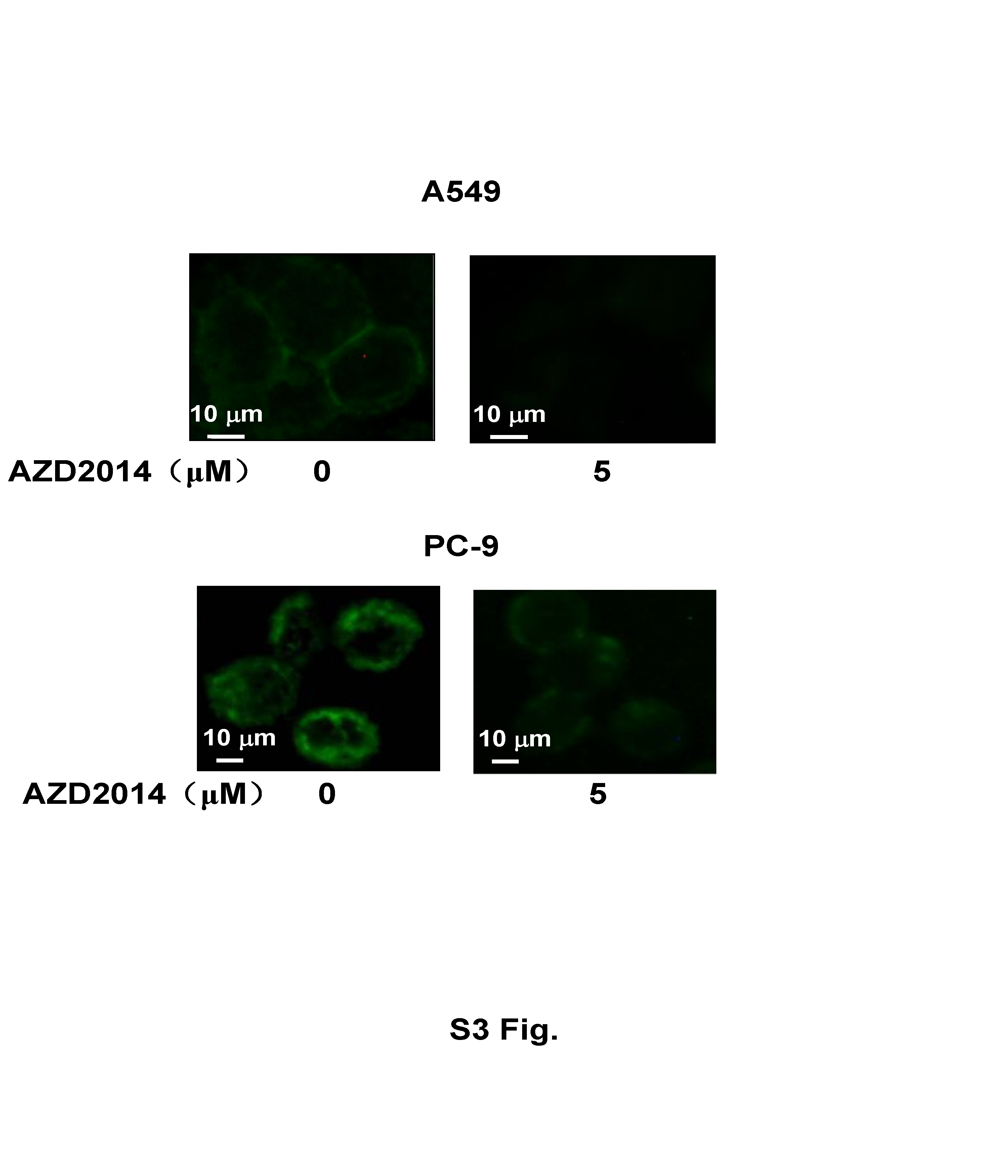

Supplement: S3 Fig — (a) Detection of GLUT1protein in A549 cells treated with 5 μM AZD2014 for 48 h by immunofluorescence assay. (b) Detection of GLUT1protein in PC-9 cells treated with 5μM AZD2014 for 48 h by immunofluorescence assay. (TIF) [file pone.0132880.s003.tif]

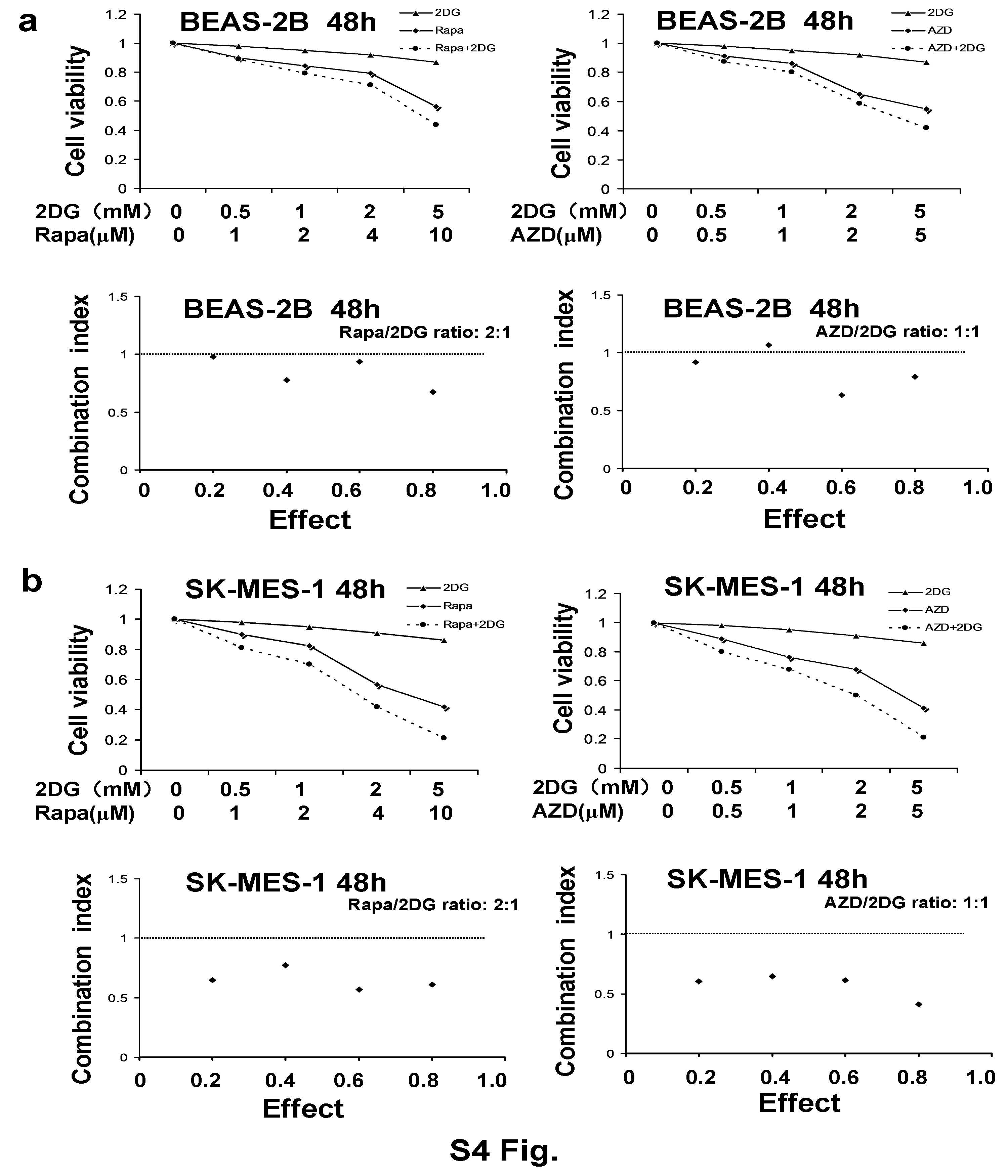

Supplement: S4 Fig — (a) Inhibitory effects of rapamycin or AZD2014 combined with 2-DG on cell proliferation in BEAS-2B cells. Cells were treated with indicated concentrations of rapamycin, AZD2014 and 2-DG for 48 h. Cell proliferation was assessed by MTT assay. (b) Inhibitory effects of rapamycin or AZD2014 combined with 2-DG on cell proliferation in SK-MES-1 cells. Cells were treated with indicated concentrations of rapamycin, AZD2014 and 2DG for 48 h. Cell proliferation was assessed by MTT assay. (a) and (b) The dose–response curve of each drug was determined and combination index (CI) values for rapamycin/2-DG concentration ratios (2:1) and for AZD2014/2-DG concentration ratios(1:1) were calculated according to the Chou–Talalay’s method at48 h time point. Diamond symbol designates the CI value for each fraction affected (effect). CI < 1, CI = 1, and CI > 1 indicate synergistic, additive and antagonistic effects, respectively. The effect ranges from 0 (no inhibition) to 1 (complete inhibition). The data are representative of three independent experiments. (TIF) [file pone.0132880.s004.tif]
